# Identification of progressors in osteoarthritis by combining biochemical and MRI-based markers

**DOI:** 10.1186/ar2774

**Published:** 2009-07-24

**Authors:** Erik B Dam, Marco Loog, Claus Christiansen, Inger Byrjalsen, Jenny Folkesson, Mads Nielsen, Arish A Qazi, Paola C Pettersen, Patrick Garnero, Morten A Karsdal

**Affiliations:** 1 Nordic Bioscience, Herlev Hovedgade 207, 2730 Herlev, Denmark; 2 University of Copenhagen, Department of Computer Science, Universitetsparken 1, 2100 Copenhagen, Denmark; 3 Delft University of Technology, Faculty of Electrical Engineering, Mathematics, and Computer Science, Mekelweg 4, 2628 CD Delft, The Netherlands; 4 Center for Clinical and Basic Research, Ballerup Byvej 222, 2750 Ballerup, Denmark; 5 CCBR-Synarc, Molecular Markers, Rue Montbrillant 16, 69003 Lyon, France

## Abstract

**Introduction:**

At present, no disease-modifying osteoarthritis drugs (DMOADS) are approved by the FDA (US Food and Drug Administration); possibly partly due to inadequate trial design since efficacy demonstration requires disease progression in the placebo group. We investigated whether combinations of biochemical and magnetic resonance imaging (MRI)-based markers provided effective diagnostic and prognostic tools for identifying subjects with high risk of progression. Specifically, we investigated aggregate cartilage longevity markers combining markers of breakdown, quantity, and quality.

**Methods:**

The study included healthy individuals and subjects with radiographic osteoarthritis. In total, 159 subjects (48% female, age 56.0 ± 15.9 years, body mass index 26.1 ± 4.2 kg/m^2^) were recruited. At baseline and after 21 months, biochemical (urinary collagen type II C-telopeptide fragment, CTX-II) and MRI-based markers were quantified. MRI markers included cartilage volume, thickness, area, roughness, homogeneity, and curvature in the medial tibio-femoral compartment. Joint space width was measured from radiographs and at 21 months to assess progression of joint damage.

**Results:**

Cartilage roughness had the highest diagnostic accuracy quantified as the area under the receiver-operator characteristics curve (AUC) of 0.80 (95% confidence interval: 0.69 to 0.91) among the individual markers (higher than all others, *P *< 0.05) to distinguish subjects with radiographic osteoarthritis from healthy controls. Diagnostically, cartilage longevity scored AUC 0.84 (0.77 to 0.92, higher than roughness: *P *= 0.03). For prediction of longitudinal radiographic progression based on baseline marker values, the individual prognostic marker with highest AUC was homogeneity at 0.71 (0.56 to 0.81). Prognostically, cartilage longevity scored AUC 0.77 (0.62 to 0.90, borderline higher than homogeneity: *P* = 0.12). When comparing patients in the highest quartile for the longevity score to lowest quartile, the odds ratio of progression was 20.0 (95% confidence interval: 6.4 to 62.1).

**Conclusions:**

Combination of biochemical and MRI-based biomarkers improved diagnosis and prognosis of knee osteoarthritis and may be useful to select high-risk patients for inclusion in DMOAD clinical trials.

## Introduction

Osteoarthritis (OA) is a slow, chronic disease characterized by cartilage degradation and typically leading to joint space narrowing (JSN), mobility loss, pain, and eventually joint replacement.

There is presently no disease-modifying osteoarthritis drug (DMOAD) with a consistent, documented effect despite several clinical attempts in late-stage phases. Some studies may have failed due to suboptimal clinical trial design [1], resulting in very low progression in placebo patients [2-4], thus reducing the power to detect potential treatment efficacy. One phase III study demonstrated a reduction of radiographic progression in the most affected knee but no effect was observed in the contralateral knee; and without reduction of pain [5]. These findings suggest that effective therapies could be developed, but also indicate the need for tools allowing identification of rapid progressors who may be suitable for inclusion in DMOADs trials.

Total joint replacement may appear to be the most valid clinical endpoint, although it is highly dependent on local health policies, patient perception, and physician assessment. Owing to the low incidence of total joint replacement, long and large studies would be needed to detect a treatment effect using this endpoint. Alternatively, an estimate of the time to surgery could be used. At present, however, no markers have demonstrated a convincing prediction of total joint replacement [6]. Additionally, such trials would probably need to target patients with end-stage disease who may not be the most adequate subjects to be studied with chondroprotective therapies.

Structural joint damage is currently monitored by JSN from plain radiographs. Since JSN has limited sensitivity to change [2,3,7], large study populations are required. Secondly, radiographs do not allow direct quantitative evaluation of cartilage tissue.

**Figure 1:**
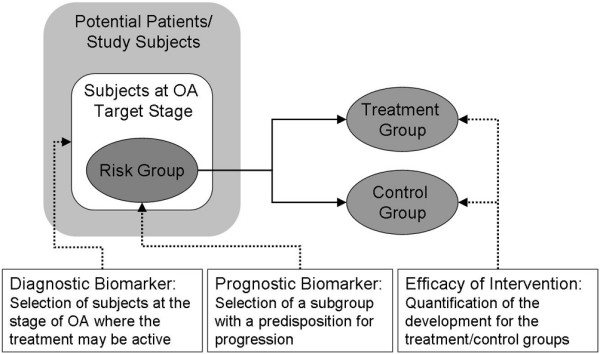
Marker types needed for clinical study. For a clinical study, diagnostic and prognostic markers are needed to select a population at the proper stage of osteoarthritis (OA) with a high risk of progression; and an efficacy marker is needed to evaluate the treatment effect. Supplementing the diagnostic marker, a burden of disease marker could be used to assess the total disease severity.

DMOAD development may be improved by appropriate biomarkers during all steps of the development process [8,9]. Several biomarker types are needed for clinical studies (Figure 1). Following the BIPED (Burden of Disease, Investigative, Prognostic, Efficacy of Intervention and Diagnostic) classification [8], a *diagnostic *marker would be useful to ensure inclusion of an homogenized population at a certain stage of the disease; and a *prognostic *marker is also needed for selecting those in this group at a high risk for disease progression. Finally, an *efficacy of intervention *marker is crucial for rapidly quantifying treatment response.

As an alternative to JSN for monitoring structural damage, biochemical markers of protease degraded cartilage matrix constituents have attracted research attention [9,10]. Some markers target pathological activities such as matrix metalloproteinase-mediated collagen type II degradation or aggrecanase-mediated aggrecan degradation [11,12]. Among them, urinary C-telopeptides of type II collagen were associated with radiographic disease risk [13,14] and with an increase in structural damage (JSN) [13]. As an example, for short proof-of-concept phase II clinical trials, the slow progression of JSN relative to the biological variation may require large study populations – here the biochemical markers may be an appealing alternative.

Alternative imaging technologies – and particularly magnetic resonance imaging (MRI) – also seem promising to assess disease progression. Specifically, MRI offers direct assessment of cartilage [15,16] and allows morphometric three-dimensional analysis. Several semi-automatic methods for cartilage quantification have been reported [17-19], including scoring systems integrating several joint features – for example, the Whole-Organ Magnetic Resonance Imaging Score [20]. Our group recently reported a fully automatic computer-based framework for quantification of several morphometric parameters, including cartilage volume, thickness, homogeneity, and curvature [21-24], targeting both cartilage quantity and quality.

Combinations of different marker modalities – for instance, markers of dynamic turnover (typically biochemical markers) and assessment of current status (for example, by MRI) – may provide complementary information and thereby superior identification of progressors for clinical trial design.

The purpose of the present study was to evaluate whether combinations of biochemical and imaging-based markers allowed, with higher accuracy than the individual markers, selection of the subjects at high risk of progression.

## Materials and methods

The radiographs, urine samples, and MRI scans for this study were acquired at baseline (BL) and at follow-up after 21 months (FU). A subgroup had BL data re-acquired for evaluating the reproducibility of the measurements.

### Population

The study included 159 subjects randomly selected to include a normal population with a large age range and a group with elevated risk of having knee OA. The majority were invited from address lists to ensure even distribution across gender and ages, supplemented with volunteers with known knee problems. The exclusion criteria ensured that no subject had previous knee joint replacement, other joint diseases (for example, rheumatoid arthritis, Paget's disease, joint fractures, hyperparathyroidism, hyperthyroidism and hypothyroidism), contraindications for performing MRI examination, or were receiving medication affecting bone and/or cartilage (for example, bisphosphonates, vitamin D, hormones, selective estrogen receptor modulators, prednisolone, anabolic androgens, and parathyroid hormone). Participants were invited to attend a follow-up visit after 21 months.

From this base collection of 318 left and right knees, five knees were excluded due to inferior imaging quality. Another 25 knees were used for training of the automatic MRI quantification methods and were excluded from the evaluation set. Furthermore, a single subject was excluded since a urine sample was not acquired. Thereby, 287 knees were included in the evaluation set at BL. A subgroup of 31 knees had imaging data re-acquired 1 week after BL. At FU, 250 knees were studied.

For each test subject, their age, sex, weight, and height were recorded at BL and FU. The baseline characteristics are presented in Table 1.

**Table 1:** Demographic and central biomarker values at baseline for the evaluation population

	Females (48%)		Males (52%)	
		
	Healthy (n = 66)	KL > 0 (n = 72)	Healthy (n = 79)	KL > 0 (n = 70)
Age (years)	47 (17)	63 (12)***	49 (16)	65 (7)***
Height (cm)	166 (6)	164 (6)*	178 (7)	176 (7)
Weight (kg)	67 (12)	72 (12)*	81 (12)	88 (12)***
Body mass index (kg/m^2^)	24.3 (4.5)	26.9 (4.2)***	25.5 (3.4)	28.4 (4.0)***
Joint space width (mm)	3.8 (0.7)	3.3 (1.2)**	4.4 (0.7)	3.3 (1.6)***
Volume (MTF.VC) (mm^3^)	5,742 (1,265)	5,906 (1,081)	8,112 (1,216)	7,468 (1,693)**
CTX-II/Cr (μg/mmol)	0.20 (0.11 to 0.36)	0.23 (0.11 to 0.48)	0.19 (0.11 to 0.32)	0.23* (0.13 to 0.41)

Demographic and central biomarker values at baseline for the 287 knees in the evaluation population (excluding the 25 knees used for training) divided by gender and by radiographic osteoarthritis status. Values presented as mean (standard deviation), or as geometric mean (± 1 standard deviation range) for the urinary collagen type II C-telopeptide marker normalized by creatinine levels (CTX-II/Cr). KL, Kellgren and Lawrence index; MTF.VC, medial tibio-femoral cartilage volume. The level of significance denotes for each gender the difference between the healthy group and the osteoarthritis group: *P < 0.05, **P < 0.01, ***P < 0.001.

Knees were scored by the Kellgren and Lawrence index (KL) [25] for the level of OA. At BL, 51% of the evaluation knees were healthy (KL 0); the overall distribution of the KL for the 287 knees scored by the KL [25] for their level of OA was [145,87,30,24,1] (for KL 0.4). For the rescan subgroup, 35% were healthy with a KL distribution of [11,13,2,5,0]. At FU 103 of the healthy individuals had remained at KL 0, and 25 individuals had progressed (defined as an increase in KL score by one or more grades). Additionally, 10 of those individuals with OA at BL had progressed at FU after 21 months (these 10 progressors were distributed [6,3,1] from KL 1 to KL 3).

All participants signed approved information consent, and the study was carried out in accordance with the Helsinki Declaration II and European Guidelines for Good Clinical Practice [26]. The study protocol was approved by the local Ethical Committee.

### Protocol and quantification for radiographs

Digital knee radiographs were acquired with the subjects standing in a weight-bearing position with knees slightly flexed and feet rotated externally. The SynaFlex (developed by Synarc, San Francisco, USA) was used to ensure position reproducibility [27].

The focus film distance was 1.0 m and tube angulation was 10° (the metatarsophalangeal view modified for fixed angle [28]). Posterior–anterior radiographs were acquired while the central beam was directed to the midpoint of the line through both popliteal regions. Radiographs of both knees were acquired simultaneously.

For each X-ray scan, the medial tibio-femoral compartment was scored by a trained radiologist. The KL was scored by qualitative evaluation of osteophytes, joint gap narrowing, and subchondral bone sclerosis for severe cases. The joint space width (JSW) was measured by manually marking the narrowest gap between the tibia and the femur. Additionally, the width of the tibial plateau was measured to quantify the knee size – covering medial and lateral compartments but excluding osteophytes. The intra-observer scan–rescan coefficients of variation were 2.5% and 0.8% for the JSW and the plateau width, respectively.

### Protocol and quantification for urine samples

For all subjects, fasting morning urine samples were collected (second void). Urinary levels of collagen type II C-telopeptide fragments (CTX-II) were measured by the CartiLaps ELISA assay (Nordic Bioscience Diagnostics, Herlev, Denmark). This assay uses a monoclonal antibody mAbF46 specific for a six-amino-acid epitope (EKGPDP) derived from the collagen type II C-telopeptide [29]. CTX-II was corrected for urinary creatinine as assessed by a standard colorimetric method. To reduce measurement and to allow precision evaluation, values were calculated as the mean of two separate determinations. For the statistical analysis, the CTX-II values were logarithmically transformed to obtain normality.

### Protocol and quantification for MRI

**Figure 2:**
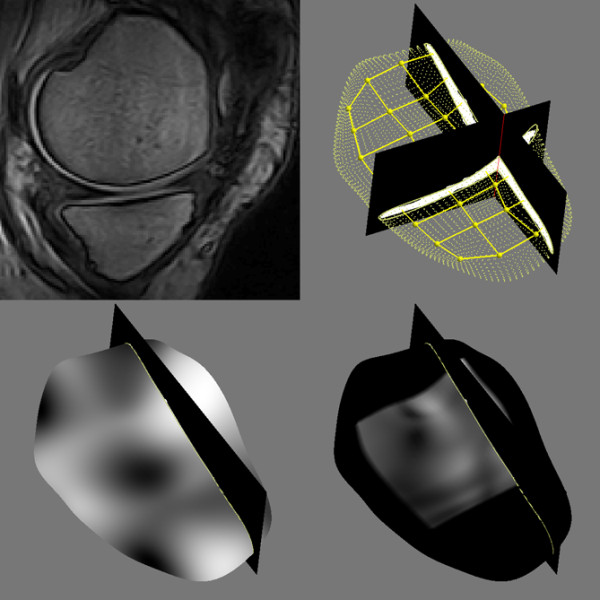
Magnetic resonance imaging-based biomarker quantification framework. Top left: a slice from a magnetic resonance imaging scan. Top right: segmentation of the medial tibial cartilage compartment shown in sagittal and coronal slice with a shape model fitted to the segmentation. Bottom left: thickness map. Bottom right: curvature map in the central region of interest used for the curvature marker. All computational steps are fully automatic.

MRI scans were acquired from a 0.18 T Esaote C-span dedicated extremity scanner (Esaote, Genova, Italy). A single knee coil was used and each knee was imaged separately. We used a sagittal Turbo 3D T1 sequence with near-isotropic voxels (40° flip angle, repetition time 50 ms, echo time 16 ms, scan time 10 minutes, resolution 0.7 mm × 0.7 mm × 0.8 mm). The scans had approximately 110 slices (depending on the knee size) and each slice was 256 × 256 pixels. Near-isotropic voxels are suitable for three-dimensional image analysis in general – and are also suitable for cartilage quantification [30]. Figure 2 (top left) shows an example MRI scan. The subjects were scanned in a supine position with no load-bearing during or prior to scanning.

The 25 scans in the training collection were segmented by slice-wise outlining of the medial tibial and femoral cartilage compartments by an expert radiologist. These segmentations were used to train a voxel classification scheme based on a multi-scale k-nearest neighbor framework [31]. This method provides automatic segmentation of the tibial and femoral cartilage compartments (Figure 2, top right).

From the segmentations, the volume and surface area were computed (MT.VC, MF.VC, MTF.VC, MT.AC, MF.AC, and MTF.AC using the Eckstein nomenclature [32]). Furthermore, the cartilage homogeneity was quantified as one minus entropy, with signal intensity entropy computed in the compartments [23] (MT.HomC, MF.HomC, MTF.HomC). Entropy quantifies the intensity histogram complexity; cartilage with more uniform intensity has lower entropy (higher homogeneity). Since the scans are T1, this measure of homogeneity is related to water distribution and proteoglycan concentration. Also, clear definition of the internal cartilage layers will be imaged by separate intensities and will contribute to higher entropy. A loss of structural integrity may therefore lead to lower entropy and higher cartilage homogeneity.

The cartilage surface roughness (inverse of smoothness) was quantified for the tibial compartment by measuring the mean surface curvature over a region-of-interest including the central load-bearing region and approximately one-half of the cartilage surface (MT.RouClAB). The surface curvature was estimated using geometric surface evolution at fine-scale resolution [21,24,33]. Fibrillation and minor focal lesions lead to decreased smoothness.

For the remaining quantifications, a statistical cartilage shape model was fitted to the segmented tibial cartilage sheets (Figure 2, top right). By training the model on healthy samples, the resulting cartilage model covers the bone area that a healthy cartilage sheet would cover [34]. The measured mean thickness thereby included denuded regions with zero thickness (MT.ThCtAB). The thickness map is illustrated in Figure 2 (bottom left). Additionally, the thickness map 10% quantile was used as a measure targeting local thinning related to focal lesions (denoted MT.ThCQ).

Finally, the mean surface curvature of the shape model was analyzed. Owing to model regularization this coarse scale curvature relates to the overall bending of the sheet and is therefore indirectly related to the congruity of the joint. This simplified congruity measure (MT.CongClAB) was quantified as the mean inverse curvature across the region of interest (Figure 2, bottom right) also used for the roughness measure [21,22,24,33].

All steps performed on the MRI are carried out in a fully automated computer-based framework in three dimensions (rather than in each individual MRI slice). The scan – rescan precision for each marker is presented in Table 2.

**Table 2:** Results for the individual and aggregate biomarkers for use as diagnostic markers and prognostic markers

Biomarker	CV (%)	Diagnostic marker			Prognostic marker			
			
		*p*_GEE_ (*n*_GEE_)	*p*_MAN_ (*n*_PA_)	AUC	*p*_GEE_ (*n*_GEE_)	*p*_MAN_ (*n*_PA_)	AUC	OR
Gender		0.55 (-)	0.6 (-)	0.53 (0.42 to 0.63)	0.46 (-)	0.49 (-)	0.56 (0.43 to 0.70)	1.8
Body mass index		0.01 (51)	0.01 (51)	0.72 (0.62 to 0.82)	0.09 (-)	0.14 (-)	0.64 (0.47 to 0.80)	2.7
Joint space width	1.8	0.002 (41)	<0.001 (36)	0.73 (0.58 to 0.86)	0.44 (-)	0.38 (-)	0.59 (0.41 to 0.78)	1.4
Width	0.7	0.13 (-)	0.21 (-)	0.62 (0.51 to 0.72)	0.2 (-)	0.46 (-)	0.57 (0.39 to 0.75)	1.1
CTX-II	11.5	0.02 (70)	0.01 (64)	0.70 (0.57 to 0.81)	0.22 (-)	0.22 (-)	0.67 (0.50 to 0.84)	3.2
Volume								
MT.VC	3.9	0.61 (-)	0.62 (-)	0.51 (0.40 to 0.63)	0.13 (-)	0.39 (-)	0.60 (0.43 to 0.76)	2.4
MF.VC	4.9	0.65 (-)	0.59 (-)	0.51 (0.38 to 0.65)	0.06 (-)	0.25 (-)	0.63 (0.49 to 0.80)	2.8
MTF.VC	3.4	0.64 (-)	0.62 (-)	0.51 (0.39 to 0.64)	0.07 (-)	0.28 (-)	0.63 (0.48 to 0.79)	2.9
Area								
MT.AC	3	0.61 (-)	0.54 (-)	0.53 (0.41 to 0.65)	0.13 (-)	0.33 (-)	0.62 (0.45 to 0.78)	2.4
MF.AC	3	0.68 (-)	0.59 (-)	0.52 (0.39 to 0.67)	0.07 (-)	0.27 (-)	0.64 (0.49 to 0.81)	1.8
MTF.AC	2.6	0.66 (-)	0.61 (-)	0.51 (0.38 to 0.64)	0.09 (-)	0.29 (-)	0.64 (0.49 to 0.80)	1.8
Thickness								
MT.ThCtAB	3.4	0.5 (-)	0.4 (-)	0.56 (0.43 to 0.67)	0.19 (-)	0.3 (-)	0.63 (0.45 to 0.80)	2.4
MT.ThCtQ	2.7	0.01 (53)	0.005 (50)	0.72 (0.61 to 0.83)	0.38 (-)	0.49 (-)	0.57 (0.40 to 0.76)	1.4
Congruity, MT.CongClAB	6.6	0.01 (52)	0.001 (37)	0.73 (0.62 to 0.84)	0.54 (-)	0.65 (-)	0.53 (0.38 to 0.69)	1.7
Roughness, MT.RouClAB	2	<0.001 (31)	<0.001 (20)	0.80 (0.69 to 0.91)	0.39 (-)	0.13 (-)	0.70 (0.54 to 0.84)	2.8
Homogeneity								
MT.HomC	0.8	0.03 (75)	0.06 (-)	0.65 (0.54 to 0.76)	0.05 (43)	0.08 (-)	0.71 (0.56 to 0.81)	3.3
MF.HomC	0.9	0.1 (-)	0.05 (106)	0.64 (0.52 to 0.76)	0.64 (-)	0.65 (-)	0.51 (0.35 to 0.68)	1.3
MTF.HomC	0.8	0.08 (-)	0.04 (94)	0.65 (0.52 to 0.76)	0.57 (-)	0.63 (-)	0.53 (0.37 to 0.69)	1.3
Longevity (basic)	1.1/0.8	0.01 (53)	0.02 (76)	0.68 (0.55 to 0.80)	0.06 (-)	0.12 (-)	0.69 (0.51 to 0.86)	4.0
Longevity-Tib	1.7/0.8	<0.001 (18)	<0.001 (16)	0.84 (0.77 to 0.92)	0.02 (30)	0.02 (32)	0.77 (0.62 to 0.90)	5.8
MRI Tib	1.5/0.8	<0.001 (20)	<0.001 (18)	0.82 (0.72 to 0.91)	0.03 (36)	0.04 (40)	0.74 (0.59 to 0.88)	4.8

Results for the individual and aggregate biomarkers for use as diagnostic markers (Kellgren and Lawrence index ≤ 1 versus >1) and as prognostic markers (early progressors versus non-progressors) evaluated in the 21-month longitudinal study with 159 subjects. Precision given as the interscan coefficient of variation (CV) for magnetic resonance imaging (MRI) quantifications and as the interscan intra-observer CV for radiograph measurements. Precision is not given for gender and body mass index since no repeated measurements were made. For the aggregate markers, precision is given for both the diagnostic/prognostic variant. Significance was estimated using the generalized estimation equations (*P*_GEE_) and multivariate analysis of variation (*P*_MAN_); the required sample size by generalized estimation equations (*n*_GEE _as number of subjects) and power analysis (*n*_PA_). Sample size estimates are excluded for non-significant markers (*P *> 0.05). Area under the receiver-operator characteristics curve (AUC) is given with 95% confidence interval. The high-risk threshold for the odds ratio (OR) was determined by cross-validation close to the median. Diagnostic and prognostic scores are median results over 500 randomly generated, representative, disjoint training/evaluation subsets. AC = cartilage area; CongClAB = cartilage congruity over the load-bearing area of bone; CTX-II = marker of collagen type II C-telopeptide fragment; HomC = cartilage homogeneity; MF = medial femoral; MT = medial tibial; MTF = medial tibio-femoral; RouClAB = cartilage roughness over the load-bearing area of bone; ThCtAB = cartilage thickness over the total area of bone; ThCQ = cartilage thickness 10% quantile; VC = cartilage volume.

### Aggregate markers of cartilage longevity

We evaluated combinations of biochemical and MRI-based markers for cartilage breakdown, quantity, and quality. Such combinations may exploit complementary information from the individual markers.

From the available markers, such a combination could be CTX-II (cartilage matrix breakdown), volume (quantity), and homogeneity (quality); we denote this aggregate marker *longevity-basic*. Here, volume and homogeneity were totals for the tibial and femoral compartments.

A more comprehensive combination includes all the available MRI quantifications. Since some quantifications were only performed in the tibial compartment, we combined CTX-II (breakdown) with all medial tibial MRI markers: volume and thickness (quantity), area (a marker of quantity; combined with volume, it may provide an aspect of quality), congruity, roughness, and homogeneity (markers for quality). We denote this aggregate marker *longevity-tib*.

Finally, for comparison, we also evaluated an aggregate marker combining all medial tibial MRI markers (that is, longevity-tib without CTX-II). This was denoted *MRI-tib*.

We investigated the performance of linear combinations of these individual markers by means of pattern recognition methods [35]. Here, methods also exist for combining markers in non-linear or non-parametric fashion [35]. We limited ourselves to combinations defined by linear discriminant analysis, however, since it allows direct interpretation of the aggregate biomarker as a weighted sum of individual markers.

### Evaluation of aggregate markers

When performing linear discriminant analysis, the resulting combination is prone to overfitting/overtraining when the number of markers is high relative to the population size, and the aggregate marker weights can be optimized to model arbitrary measurement variations that are not representative of the actual disease progression.

We therefore performed an evaluation where the population was repeatedly split randomly into two subpopulations with approximately equal size and distribution of levels of OA. For each split, we optimized the weights for the aggregate biomarker on one training subpopulation (using linear discriminant analysis) and we evaluated the resulting aggregate marker on the other evaluation subpopulation. The median performance on the evaluation subpopulations estimates the aggregate marker performance including generalization ability. We used 500 repetitions.

In order to allow direct comparison of individual and aggregate markers, we evaluated the individual markers equivalently using repeated random subpopulations.

### Statistical analysis

The demographic and biochemical markers provide one measurement per subject. The markers based on radiographs and MRI scans each provide one measurement per knee. This requires specific handling of the intra-subject correlation between knee observations in the analysis. We perform this in two alternative ways in the analysis. Firstly, we combine the two knee measurements into a single subject measurement by averaging – this allows use of standard statistical analysis. Secondly, we perform analysis by generalized estimation equations (GEE) that explicitly model the inter-knee correlation within subjects.

We defined the diagnostic performance as the ability of the BL marker values to separate healthy or borderline cases (KL ≤ 1) from OA knees (KL >1). For the subject-averaged measurements this was evaluated by the *P *value from multivariate analysis of variation (based on Hotelling's *T*^2 ^test [36]), by the corresponding required study population size calculated from power analysis (*n*_PA_) requiring 80% power and a significance level of 0.05, and by the area under the receiver-operator characteristics curve (AUC). We used DeLong and colleagues' non-parametric approach [37] to test whether AUC values were statistically different. Using GEE we also calculated the *P *value and the sample size (*n*_GEE_), again requiring 80% power and a significance of 0.05. The GEE *P *value was computed using the GEEQBOX package [38], and the sample size was calculated by a Matlab implementation of Rochon's procedure [39].

The prognostic performance was defined as the ability of the BL values to separate healthy non-progressors (KL 0 at BL and FU) from early progressors (KL 0 at BL and KL > 0 at FU), and was evaluated by the same analysis as for diagnostic markers above and then adding the odds ratio (OR). For estimating the OR, the population was split into low/high groups where the threshold for each marker was defined by cross-validation on the train/evaluation subpopulations (unless explicitly stated otherwise). The Breslow-Day test using Tarone's adjustment [40] was used for testing whether differences between ORs were statistically significant. Analysis of progression at other KL levels was not performed due to the low number of progressors.

The choices of the AUC and OR as evaluation parameters for diagnostic and prognostic markers follows the BIPED classification [8].

The potential confounding effects of gender, age, and body mass index were investigated by application of linear correction to the key aggregate markers.

## Results

The diagnostic and prognostic abilities of individual and aggregate markers are presented in Table 2.

JSW performed well as a diagnostic marker (AUC = 0.73) – as expected, since it is part of the KL score. The best individual diagnostic marker was cartilage roughness (AUC = 0.80, *n*_GEE_*/n*_PA _= 31/20). The cartilage longevity marker also demonstrated good performance (AUC = 0.84, *n*_GEE_*/n*_PA _= 18/16). The AUC for longevity-tib was statistically significantly higher than for all individual markers (*P *< 0.05).

Several individual markers demonstrated prognostic ability, among these CTX-II (AUC = 0.67, OR = 3.2), cartilage roughness (AUC = 0.7, OR = 2.8), and cartilage homogeneity (AUC = 0.71, OR = 3.3). The JSW seemed inappropriate as a prognostic marker (*P *= 0.4). Cartilage longevity-tib also performed well as a prognostic marker (AUC = 0.77, OR = 5.8, *n*_GEE_*/n*_PA _= 30/32). The OR for the longevity marker was significantly higher than for all individual markers (*P *< 0.05) except for roughness and homogeneity (*P *= 0.2 and *P *= 0.3). The AUC was higher (*P *< 0.05) except for homogeneity (*P *= 0.12).

### Cartilage longevity markers

When the individual markers are rescaled to have a standard deviation of one (denoted by underlining), the aggregate marker weights give an estimate of the marker importance. As examples, the diagnostic and prognostic cartilage longevity-tib markers (Vol: MT.VC, Area: MT.AC, Thick: MT.ThCtAB, Cong: MT.CongClAB, Rough: MT.RoughClAB, Hom: MT.HomC) were:





Below we present further results for these aggregate cartilage longevity-tib markers.

**Figure 3:**
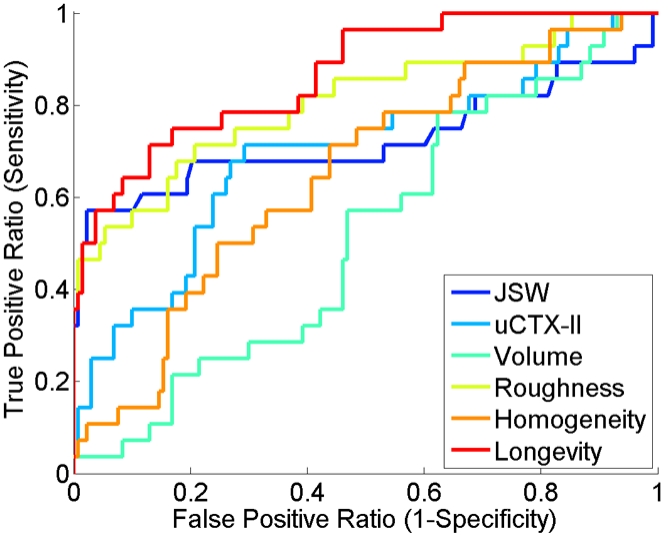
Diagnostic ability for separating healthy individuals from osteoarthritis subjects. The diagnostic ability for separating healthy individuals from osteoarthritis (OA) subjects (defined by Kellgren and Lawrence index >1) of key markers, illustrated by a receiver-operator characteristics diagram. The areas under the curves are: joint space width (JSW), 0.73; urinary marker of collagen type II C-telopeptide fragment (uCTX-II), 0.70; volume, 0.52; roughness, 0.81; homogeneity, 0.65; and longevity-tib, 0.87. The aggregate longevity-tib marker provided superior ability to all the individual markers (*P *< 0.05).

**Figure 4:**
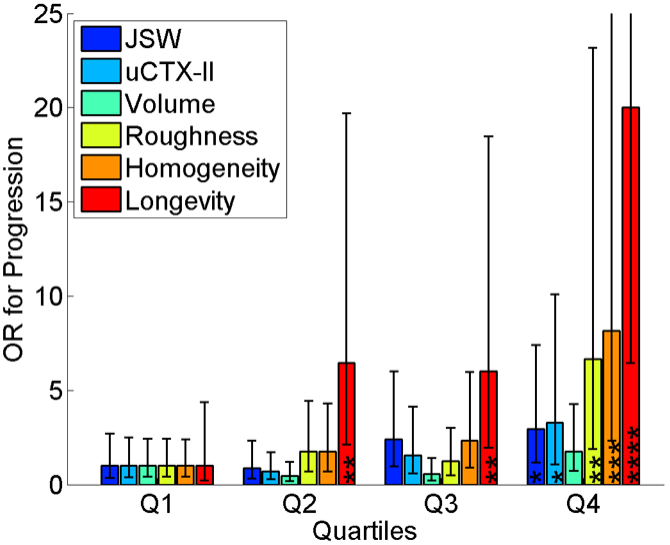
Prognostic ability of key markers for separating healthy non-progressors from early progressors. Early progressors were defined by whether the KL score increased from a baseline score of 0. For each marker, the population was divided into quartiles and each quartile was compared with the lowest quartile in terms of the odds ratio (OR) for predicting the progressors. Each OR is given with the 95% confidence interval and with the significance level: *P < 0.05, **P < 0.01, ***P < 0.001, and ****P < 0.0001. Cartilage longevity-tib proved superior to the individual markers (*P *< 0.05) except for roughness/homogeneity (*P *= 0.2/0.3) with OR of 20.0 for the highest quartile. JSW = joint space width; uCTX-II, urinary marker of collagen type II C-telopeptide fragment.

These aggregate markers are compared with the key individual markers in Figures 3 and 4. The receiver-operator characteristics curves in Figure 3 show that both the JSW and longevity were able to diagnose 57% true positives with 3.8% false positives. From there, the longevity marker proved better at diagnosing the borderline cases. The AUC for longevity was 0.87, which was superior to the AUC for a JSW of 0.73 (*P *= 0.02) and the AUC of 0.81 for the best individual marker roughness (*P *= 0.02).

Figure 4 elaborates on the prognostic performance. For each marker the scores were split into quartiles and the predictive power of elevated scores were computed by comparison with the lowest quartile. The highest quartile of the cartilage longevity marker provided an OR of 20.0 (95% confidence interval = 6.4 to 62.1).

### Gender, age, and body mass index adjustment

When adjusting the longevity markers for gender, age, and body mass index, the diagnostic marker retained performance very similar to the unadjusted (AUC = 0.83, *n*_PA _= 17). The prognostic longevity marker also retained equivalent performance (AUC = 0.77, OR = 5.8, *n*_PA _= 28).

### Markers normalized to knee size

In previous work, we used MRI cartilage markers normalized by the width of the tibial plateau to adjust for joint size. This improved diagnostic performance for the markers [22] and can also be used in the aggregate markers [41]. Using normalized MRI markers [22], both the diagnostic longevity marker (AUC = 0.84, *n*_GEE_*/n*_PA _= 21/16) and the prognostic longevity marker (AUC = 0.75, OR = 4.8, *n*_GEE_*/n*_PA _= 28/39) retained very similar performance as the non-normalized markers.

### Diagnosis at Kellgren and Lawrence index above zero

Above, the diagnostic markers are evaluated for the ability to separate KL ≤ 1 from KL >1. In order to target diagnosis of very early OA, the separation could be KL = 0 from KL > 0. On comparing with the markers in Table 2, the best individual diagnostic markers are then the JSW (AUC = 0.70), congruity (AUC = 0.71), and homogeneity (MT.HomC, AUC = 0.70). The cartilage longevity marker allowed improved performance (AUC = 0.82, *n*_GEE_*/n*_PA _= 21/21).

### Prediction of joint space narrowing and cartilage loss

The aggregate prognostic markers were optimized to predict progression in the KL score. The same prognostic longevity marker, however, also predicts increased longitudinal JSN and cartilage loss. Specifically, when dividing the knees into those above/below the mean longevity score, the mean JSN is 4.9 percentage points higher (*P *= 0.11), the mean tibial + femoral cartilage loss is 2.5 percentage points higher (*P *= 0.10), and the mean femoral cartilage loss is 2.6 percentage points higher (*P *= 0.05) for the high-risk group.

## Discussion

The complexity of OA makes biomarker development challenging. There are many onset factors including genetics, trauma, biomechanics, weight, and exercise; and different phases of OA may entail different pathological mechanisms. Biomarkers therefore can target numerous effects, including increased turnover in cartilage and bone, fibrillation, subchondral bone thickening, bone edema, osteophytes, focal cartilage lesions, and eventually cartilage denudation (see models of OA stages [42,43]). Owing to the heterogeneity of the disease, numerous effects will be observable concurrently in a population, and therefore aggregate markers may allow more comprehensive quantification in clinical studies.

We evaluated diagnostic and prognostics markers combining a urine-based biochemical marker for cartilage breakdown with MRI-based markers of cartilage quantity and structure. Markers combining the quantity, quality, and current breakdown could conceivably be comprehensive markers for cartilage longevity.

The major findings were twofold. The best individual diagnostic marker was cartilage roughness (AUC = 0.80, *n*_GEE _= 31) and the best individual prognostic marker was homogeneity (AUC = 0.71, *n*_GEE _= 43). Secondly, the aggregate cartilage longevity-tib marker (combining CTX-II, volume, area, thickness, congruity, roughness, and homogeneity) performed well diagnostically (AUC = 0.84, *n*_GEE _= 18) and prognostically (AUC = 0.77, OR = 5.8, *n*_GEE _= 30). The performance persisted after adjustment for gender, age, body mass index, and knee size.

### Presently accepted marker

The results demonstrated that use of the JSW for population selection in clinical studies may not be optimal. The JSW was unsuitable as a prognostic marker and the diagnostic performance (AUC = 0.73) is expected since the JSW is integrated in the definition of OA (KL). Even so, roughness has a higher AUC (0.80, *P *< 0.05). When inspecting Figure 3, it is apparent that the JSW is effective in diagnosing the severe cases (left end of curves) corresponding to low JSW. For the earlier stages of OA, however, homogeneity and in particular cartilage longevity-tib outperforms the JSW.

### Scalability for large, multicenter studies

Aggregate markers combining several individual markers introduce a potential measurement bottle-neck. Even for volumetric MRI markers, manual/semi-automatic annotation is time consuming. For advanced three-dimensional markers (such as curvature or roughness), manual annotation is not feasible.

The present study relied on fully automated computer-based MRI methods for cartilage status assessment and a standardized biochemical marker measured through standard ELISA techniques. The presented aggregate markers can thereby be applied in large, multicenter studies without introducing a reader bottle-neck.

### Aggregate markers

The cartilage longevity markers support the hypothesis that markers from different modalities can be complementary. Even with similar markers, superior combined performance could be achieved by improved precision through repeated similar quantifications. The cartilage longevity-tib marker has precision 1.7/0.8%. For comparison, cartilage homogeneity has precision 0.8%. The improved performance is therefore probably due to the combination of the complementary aspects of cartilage quantity, quality, and breakdown measured from different modalities.

A potential extension of the presented methodology is to include additional complementary MRI markers targeting bone, meniscus, and other joint structures; and to include additional biochemical markers reflecting bone turnover, synovitis, cartilage formation, cartilage degradation mediated by biological processes of type II destruction different from CTX-II [44], or destruction of other matrix proteins, such as aggrecan. The aggregate markers could thereby become more similar to composite markers such as the Whole-Organ Magnetic Resonance Imaging Score [20] and the Knee Osteoarthritis Scoring System [45] MRI scoring methods. These scoring systems provide semiquantitative scores by inspection of MRI for presence/severity of disease-related parameters (for example, cartilage lesions, bone marrow abnormalities, and meniscal abnormalities). For such comprehensive aggregate markers, automatic MRI analysis will be even more important to minimize the expert reader burden.

### Limitations of the study

We focused the investigation of progression of OA to the early stages. Specifically, we focused on the subpopulation with early radiographic signs of OA at baseline (KL <2). The conclusions are therefore only valid for progression during the early stages of OA. A study population with more progressed OA would be needed to validate the findings at later stages of OA. Furthermore, the relatively small number of subjects in the present study implies that the findings need to be validated on larger populations.

Furthermore, validation on larger populations is also needed to determine specific threshold values for the different markers – for example, for determining the high-risk population. In addition, the somewhat complicated nature of aggregate markers implies that validation on several populations is needed to facilitate the clinical interpretation and confidence in the markers.

The cartilage measurements were based on an MRI scanner with a 0.18 T magnet. The use of low-field MRI is sparsely validated compared with high-field MRI [46]. In particular, high-field MRI may allow cartilage volume measurements with higher accuracy and precision (implying that studies may be conducted with smaller populations). Low-field MRI, however, is much cheaper and easier to install and maintain. Future studies are needed to evaluate whether low-field MRI can be a cost-effective alternative to high-field MRI for clinical studies.

The study used the common KL score as the definition of OA. This score is not compartment specific or feature specific, whereas the markers were both compartment specific (MRI), joint specific (JSW), and not joint specific (CTX-II). Future studies are needed to elucidate the relationships between specific features and specific compartments – for example, studies similar to that of Blumenkrantz and colleagues [47].

## Conclusions

Owing to the complexity of OA, it is unlikely that any single marker will be suitable for all stages of the disease. The different biomarker modalities, however, may offer complementary information, which suggests that aggregate markers may provide superior biomarker performance.

In the present study we evaluated markers from urine samples, radiographs, and MRI scans. The results demonstrated that aggregate markers may indeed provide superior diagnostic and prognostic markers; the proposed cartilage longevity marker combining aspects of cartilage quantity, quality, and breakdown performed well both as a diagnostic and a prognostic marker.

The proposed aggregate marker methodology may therefore have a direct impact on clinical study design. By allowing selection of a high-risk population, the study sample size can be lowered while still improving the chance of a positive study outcome. This should facilitate the development of effective DMOADs.

## Abbreviations

AC: cartilage area; AUC: area under the receiver-operator characteristics curve; BIPED: Burden of Disease, Investigative, Prognostic, Efficacy of Intervention and Diagnostic; BL: baseline; CongClAB: cartilage congruity over the load-bearing area of bone; CTX-II: marker of collagen type II C-telopeptide fragment; DMOAD: disease-modifying osteoarthritis drug; ELISA: enzyme-linked immunosorbent assay; FDA: US Food and Drug Administration; FU: follow-up; GEE: generalized estimation equations; HomC: cartilage homogeneity; JSN: joint space narrowing; JSW: joint space width; KL: Kellgren and Lawrence index; MF: medial femoral; MRI: magnetic resonance imaging; MT: medial tibial; MTF: medial tibio-femoral; *n*_GEE_: required study population size calculated from GEE; *n*_PA_: required study population size calculated from power analysis; OA: osteoarthritis; OR: odds ratio; RouClAB: cartilage roughness over the load-bearing area of bone; ThCtAB: cartilage thickness over the total area of bone; ThCQ: cartilage thickness 10% quantile; VC: cartilage volume.

## Competing interests

EBD and IB are employees of Nordic Bioscience. MN is partly funded by Nordic Bioscience. CC and MAK are employees and shareholders of Nordic Bioscience. PCP is employed by the Center for Clinical and Basic Research (CCBR). JF and AAQ have both received scholarships partly funded by Nordic Bioscience. ML was previously partly funded by Nordic Bioscience. PG is employed by CCBR-Synarc. The study was sponsored by CCBR and Nordic Bioscience. The commercial rights to the software used for automatic cartilage quantification from MRI are held by Nordic Bioscience. A patent for the proposed Longevity markers is pending.

## Authors' contributions

All authors contributed to the discussion leading to the study and the writing of the manuscript. In particular, the marker combination methodology was developed by EBD and ML. The statistical analysis was designed and carried out by EBD and IB. The MRI analysis methods were developed by JF, AAQ, MN, and EBD. The radiological reading was performed by PCP. The biochemical marker expertise and measurements were provided by IB, CC, MAK, and PG. All authors read and approved the final manuscript.
